# Effect of rosemary essential oil and modified-atmosphere packaging (MAP) on meat quality and survival of pathogens in poultry fillets

**DOI:** 10.1590/S1517-838246220131201

**Published:** 2015-06-01

**Authors:** Tolga Kahraman, Ghassan Issa, Enver Baris Bingol, Beren Basaran Kahraman, Emek Dumen

**Affiliations:** 1 Istanbul University, Department of Food Hygiene and Technology, Faculty of Veterinary Medicine, Istanbul University, Istanbul, Turkey, Department of Food Hygiene and Technology, Faculty of Veterinary Medicine, Istanbul University, Istanbul, Turkey.; 2 Culinary Program, Avrupa Vocational School, Istanbul, Turkey, Culinary Program, Avrupa Vocational School, Istanbul, Turkey.; 3 Istanbul University, Department of Microbiology, Faculty of Veterinary Medicine, Istanbul University, Istanbul, Turkey, Department of Microbiology, Faculty of Veterinary Medicine, Istanbul University, Istanbul, Turkey.

**Keywords:** rosemary, essential oil, modified-atmosphere packaging, poultry, pathogens

## Abstract

The effect of rosemary (*Rosmarinus officinalis L.*) essential oil (REO) and modified-atmosphere packaging (MAP) on the survival of certain pathogens (*Salmonella* Typhimurium and *Listeria monocytogenes*) in poultry fillets and on their meat quality during 7 days of refrigerated storage were investigated. Because REO at 0.05% and 0.1% had weak antibacterial activity and REO at 0.3%, 0.5% and 1.0% imparted unacceptable organoleptic properties, only REO at 0.2% was used to treat the poultry meat. The results showed that adding 0.2% REO to poultry fillets did not reduce the size of the population of *S.* Typhimurium and *L. monocytogenes.* However, REO treatment significantly decreased the L* (lightness) value and increased the a* (redness) value of stored fillets, and adding REO in combination with MAP reduced the level of lipid oxidation. In conclusion, in a suitable combination, REO can be applied to improve the quality of meat, but further studies should be conducted to determine the appropriate commercial level for different meat products.

## Introduction

The production of poultry meat products has increased throughout the world due to its specific sensory attributes and the consumer's belief that white meat is healthier than red meat. Poultry products are highly perishable foods. Therefore, the industry is focused on methods to increase the shelf-life and the overall safety and quality of poultry products (Geornaras *et al.*, 1995; Colak *et al.*, 2011).

The hygienic quality of poultry products depends on the personal hygiene of the handlers, the production method, the qualities of all of the ingredients and the raw meat used (Colak *et al.*, 2011). Many researchers have reported that poultry meat and its products were contaminated by several pathogenic bacteria (Basaran Kahraman and Ak, 2012; Cetinkaya *et al.*, 2008; Mataragas *et al.*, 2008; Urumova *et al.*, 2014). Consumption of products prepared using contaminated poultry meat has been found to be the cause of large outbreaks of salmonellosis and listeriosis (EFSA, 2012). The causative agents of these foodborne pathogens can be present in the gastrointestinal tracts of food-producing animals and can subsequently be transferred to humans through the production, handling and consumption of meat and meat products (Norrung and Buncic, 2008). Salmonella infections were reported to cause approximately 1.4 million cases of foodborne illness and more than 500 deaths per year in the USA (Cetinkaya *et al.*, 2008). In 2010, 99020 cases of human salmonellosis were reported in the EU countries, whereas there were 1,601 cases of human listeriosis in the EU countries that year (EFSA, 2012). To solve these serious food safety problems for consumers, producers are seeking new and improved manufacturing practices to prevent microbiological contamination, survival and growth (Bingol *et al.*, 2013).

Consumers are increasingly focused on the use of natural products rather than synthetic additives in foods. This trend was reflected in the recently issued EU Directive 2006/52/EC (EU 2006) referring to the necessity of reducing the use of nitrites in processed food. The largest groups of natural compounds are the essential oils and plant extracts. Essential oils are aromatic and volatile oily liquids that are obtained from plant materials, such as leaves, fruits, seeds, and fruits (Gutierrez *et al.*, 2008).

Among the essential oils, rosemary (*Rosmarinus officinalis L.*) oil is widely used for preservation by the food industry. Studies have demonstrated the beneficial effects of rosemary essential oil (REO) as a natural antioxidant that prevented color deterioration and/or lipid oxidation (Aruoma *et al.*, 1992; Balentine *et al.*, 2006; Hussain *et al.*, 2010; Yu *et al.*, 2002). In addition to its strong antioxidative properties, REO was shown to inhibit the growth of various foodborne pathogens in vitro (Hammer *et al.*, 1999; Smith-Palmer *et al.*, 1998); however, its effectiveness when applied to food has not been extensively investigated.

The practical application of several essential oils in foods is limited due to the strong flavor they impart and their interaction with some food ingredients. The preservative effect of essential oils and extracts may be achieved by using them at lower concentrations in combination with other preservation strategies, such as modified-atmosphere packaging (MAP) (Mastromatteo *et al.*, 2010). MAP has gained considerable acceptance as a modern method for food preservation. MAP is a well-known method for extending the shelf-life of a variety of foods, including poultry and fresh meat (Davies 1995).

The aim of this study was to determine the effect of REO and MAP on the survival of certain pathogens (*S.* Typhimurium and *L. monocytogenes*) in poultry fillets and on their meat quality.

## Materials and Methods

### Extraction of REO

Leaves collected from rosemary (*Rosmarinus officinalis L.*) plants in the Aegean region of Turkey were air dried at room temperature (20 ± 2 °C), and their essential oils were obtained by a 3-h continuous steam distillation using a Clevenger-type apparatus. The essential oil was collected, dried over anhydrous sodium sulfate and stored at 4 °C until analysis.

### Gas Chromatography

A 0.4-μL aliquot of essential oil was subjected to analysis using capillary gas chromatography. A Thermo Finnigan Trace GC Ultra system (Thermo Electron Corporation, Milan Italy) equipped with an HP-5MS capillary column (30 m x 0.25 mm) with a 0.25-μm film thickness was used for this study. The detector and injector were maintained at 275 °C and 200 °C, respectively. Helium was used as the carrier gas, at a flow rate of 1.0 mL/min, and the split ratio was set to 100:1. The column temperature was programmed as follows: isothermal at 60 °C for 1 min, ramp to 300 °C at 3 °C/min and isothermal for 1 min. The constituents were identified by comparing their mass spectra with those in the computer library and those of authentic compounds. The identifications were confirmed by comparing their retention times (RT) with those of authentic compounds (1.8-cineole, α-pinene, camphor, 2α-pinene, camphen and caryophyllene).

### Mass spectrometric analysis

The chemical composition of the REO was determined from gas chromatography-mass spectrometry (GC-MS) analysis. Thermo Finnigan Trace DSQ system (Thermo Electron Corporation, Milan Italy), equipped with an HP-5MS capillary column (30 m x 0.25 mm) with a 0.25-μm film thickness was used. The chromatographic conditions were identical to those used for the gas chromatographic analysis.

### Culture preparation

Samples of the *S.* Typhimurium (ATCC 14028) and *L. monocytogenes* (ATCC 7644) strains were obtained from Microbiologics® (Minnesota, USA). Both strains were stored in glycerol (30%) at −80 °C. Samples were streaked on Tryptone Soya Agar (Oxoid CM131, Basingstoke, England) plates and were incubated at 35 °C overnight. After 24 h, *S.* Typhimurium and *L. monocytogenes* were grown in aerobically Tryptone Soya Broth (TSB; Oxoid CM129) at 37 °C until used.

### Assays for antibacterial activity

The antibacterial activity of the REO was assayed as follows: samples of both bacterial cultures (10^8^ cfu/mL) were inoculated into 10 mL of TSB. Appropriate amounts of the REO solution were added to TSB to achieve final concentrations of 0.05%, 0.1%, 0.2%, 0.3%, 0.5% and 1.0% (w/v). Broth samples of all the tested treatments were incubated at 37 °C for 24 h. To determine the inhibitory effects of REO, samples of the inoculated broth were taken for microbiological analysis at 0, 4, 8, 12 and 24 h of incubation; all of the analyses were performed in duplicate. The antibacterial activity was determined from the growth of viable *S.* Typhimurium and *L. monocytogenes* cells on Xylose Lysine Deoxycholate Agar (Oxoid CM469) or Chromogenic Listeria Agar (Oxoid CM1080) for (Solomakos *et al.*, 2008), respectively.

### Inoculation of the poultry fillets

The *S.* Typhimurium and *L. monocytogenes* strains were individually prepared by growing samples in 10 mL of TSB at 30 °C for 24 h. The bacterial strains were sub-cultured twice using TSB before use. The bacteria were centrifuged (8000 x *g*) at 4 °C for 10 min, washed using sterile phosphate-buffered saline (PBS) and serially diluted using PBS to a concentration capable of providing approximately 10^4^ cfu/g of poultry samples.

Poultry breast meat (totally 21 kg) was obtained from a poultry (broiler) processing plant within 12 h of slaughter. Immediately after delivery, the meat was filleted in small pieces (20 g). The poultry fillets were divided into three equal groups, and each portion was placed in a polyethylene bag. The samples in the first group were contaminated with only *S.* Typhimurium and those in the second group were contaminated with only *L. monocytogenes*. The samples in the non-inoculated group were used for physicochemical and sensorial analyses. The samples of poultry fillets were placed in stomacher bags and were inoculated with a single pathogenic strain. The inoculated samples were manually massaged for 10 min at room temperature (20 ± 2 °C) to ensure proper distribution of the pathogens. Prior to inoculation, the fillets were examined for contamination by the tested pathogens.

Following homogenization, the inoculated and non-inoculated samples (*S.* Typhimurium, *L. monocytogenes* and no bacterial inoculum) were treated using four different methods. The treatments were (1) air packaging (2) air packaging + the addition of REO at 0.2%, (3) MAP, (4) MAP + the addition of REO at 0.2%. REO levels of 0.05% and 0.1% were not further tested due to their weak antibacterial activities against the selected pathogens. In addition, 0.3%, 0.5% or 1.0% REO solutions were not applied in the experimental design because of the unacceptable organoleptic properties they imparted to the poultry meat.

Immediately after treatment, all of subgroup samples (300–350 g) were placed in low-O_2-_permeable (8–12 cm^3^/m^2^/24 h at STP) polystyrene/ethylvinylalcohol (EVOH)/polyethylene (PE) trays. For air packaging, the PE trays were over-wrapped using non-barrier polyvinylchloride (PVC) cling film but not sealed to allow exposure to the atmospheric air. For MAP, the PE trays were over-wrapped using oxygen-permeable (6000–8000 cm^3^/m^2^/24 h at STP) polyvinyl-chloride film (Wrap Film Systems Ltd., Shropshire, England) into which a gas mixture of 30% CO_2_/70% N_2_ was injected using a Ponapack packaging machine (VTK 40 SC, Ponapack, Istanbul, Turkey). The packages were stored at 4 °C and were analyzed at 0, 1, 3, 5 and 7 days to determine the microbiological, physico-chemical (pH, TBA, instrumental color) and sensorial (color, odor, taste and flavor) characteristics, using six packages from each group on each sampling date.

### Gas analysis of the package atmospheres

Gas analyses of the atmosphere within the packages were conducted in duplicate at 1, 3, 5 and 7 days of storage. The CO_2_, O_2_ and N_2_ contents within the packages were determined by injecting 0.5 mL of gas that had been removed from the headspace using a syringe (B. Braun, Melsungen, Germany) into a PDI gas chromatography system (PBI-Dansensor A/B, Ronnedevaj 18, Ringsted, Denmark) fitted with a thermal-conductivity detector. The headspace gas levels were calculated by subtracting the O_2_% and CO_2_% after a single direct reading of each package's atmosphere.

### Microbiological analysis

Samples (25 g) were added to 225 mL of buffered peptone water (Oxoid CM509) in sterile stomacher bags (Seward, Worthing, England) and were homogenized for 2 min using a stomacher device (Interscience, St. Nom la Breteche, France). Following homogenization, 10-fold serial dilutions were prepared using sterile the Maximum Recovery Diluent (Oxoid CM317), and the diluted samples (0.1 mL) were streaked onto Xylose Lysine Deoxycholate Agar (Oxoid CM469) and Chromogenic Listeria Agar (Oxoid CM1080) supplemented with Listeria Selective Supplement (Oxoid SR227) and Listeria Differential Supplement (Oxoid SR228) for enumeration of the *S.* Typhimurium and *L. monocytogenes*, respectively (Harrigan, 1998; Hitchins, 1995). The microbiological analyses were conducted in triplicate.

### Physicochemical analysis

The pH value of each poultry sample was determined after each exposure period by blending it with 100 mL of distilled-deionized water (ddH_2_O) and measuring the value using a pH meter (Hanna HI 1131, Germany) equipped with a combined electrode (HI 9321 Microprocessor pH meter, Hanna Instruments, Germany) (AOAC 1984).

The color of each sample was measured using a Colorflex HunterLab spectrophotometer (Hunter Associates Laboratory Inc., Reston, VA, USA). Before each measurement, the apparatus was calibrated using a white, a black and a reference standard. Color coordinate values, including the L* (lightness), a* (redness), and b* (yellowness) values were determined based on the average of five readings that were performed at different locations on the surface of the sample. The color was evaluated using diffuse illumination (D65 2° observer) with an 8-mm viewing aperture and a 25-mm port size, with the specular component excluded (Hunt *et al.*, 1991).

The thiobarbituric acid (TBA) content was determined according to the method described by Pearson *et al.* (1991). The absorbance at 538 nm was measured using a T80+ UV/VIS spectrometer (PG Instruments Ltd., London, UK). The TBA content was expressed as mg of malondialdehyde (MDA) and was calculated by multiplying the absorbance values by the standard (K) value.

### Sensory evaluation of poultry fillets

The sensorial attributes of the poultry fillets were evaluated by eight experienced panelists ranging from 26 and 45 years of age (2 females and 6 males) who were trained according to the ISO protocol (1993). Prior to performing the analysis, the panelists were taught the vocabularies of the sensory attributes (color intensity, taste and flavor: flavor intensity, spicy taste, salty taste, sweet taste, acidic taste and odor: odor intensity, sour odor, sweet odor, spicy odor) using a standardized procedure (ISO 1998) in two separate sessions of approximately 2 h for each of the selected attributes, which was followed by an open-discussion session to familiarize the panelists with the attributes and the scales they would use.

The panelists evaluated the perceived intensity of each sensory attribute on 10-cm long unstructured linear scales that were verbally anchored at each end. Simultaneously, the overall acceptability of the poultry meat was assessed using a 5-point descriptive scale in which 1 referred to "dislike extremely" and 5 referred to "like extremely".

The panel members were seated in individual booths in a temperature- and light-controlled room (fluorescent lighting at 2000 lx; Philips 40-W Cool White bulbs) and were given a set of six samples in a completely randomized order. Before the evaluations were performed, the poultry samples were removed from the packages and wrapped in aluminum foil and then were individually cooked in an oven (at 220 °C) for 20 min. Each of the samples was served warm in a dish encoded using a three-digit number. Unsalted crackers and water were served to the panelists to freshen their mouths between assessing each sub-sample. The sensory evaluations of the fillets were performed at 1, 3, 5 and 7 days of refrigerated storage at 4 °C.

### Statistical analysis

An analysis of variance was conducted for each variable to investigate the effect of the antibacterial activities of REO during the storage period. The trials were performed in triplicate, and the General Linear Model procedure (PROC GLM) of the SPSS 13.0 package was used to analyze the data (SPSS 2001), with significant differences defined as p < 0.05. The microbial counts were expressed as log cfu/g and the mean differences were determined using Duncan's multiple-range tests.

## Results and Discussion

The main volatile components of the REO that was used to treat the poultry fillets were characterized according to the contents of the prominent (> 1%) components, including those of 1.8-cineole (44.80%), α-pinene (13.17%), camphor (10.43%), 2α-pinene (8.08%), camphen (5.16%) and caryophyllene (5.07%). In the present study, 1.8-cineole and α-pinene were found to be the major constituents of the REO. Similar results were found in previous studies (Jiang *et al.*, 2011; Pintore *et al.*, 2002). Boutekedjiret *et al.* (1999) reported that the main component of Tunisian, Turkish and Italian REOs was 1.8-cineole, which accounted for more than 40% of the total contents. The composition of an REO is affected by the plant variety, the geographical region of cultivation and the extraction method used (Burt 2004).

**Figure 1:**
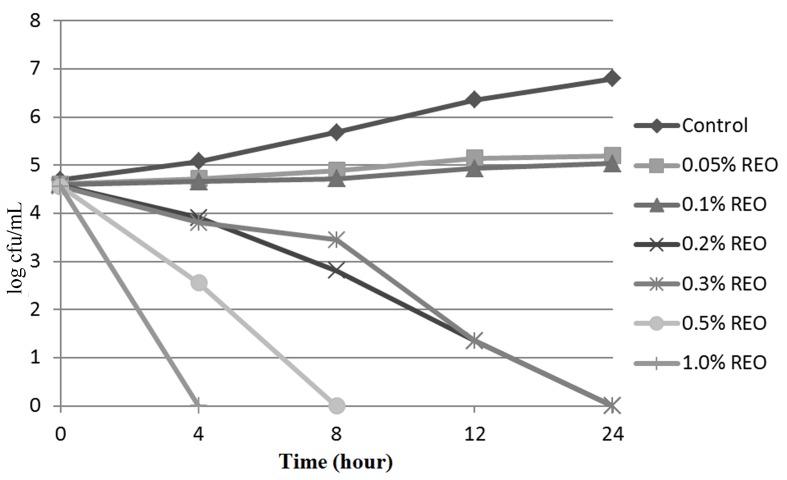
Effect of the REO on the survival of *S.* Typhimurium in TSB.

**Figure 2:**
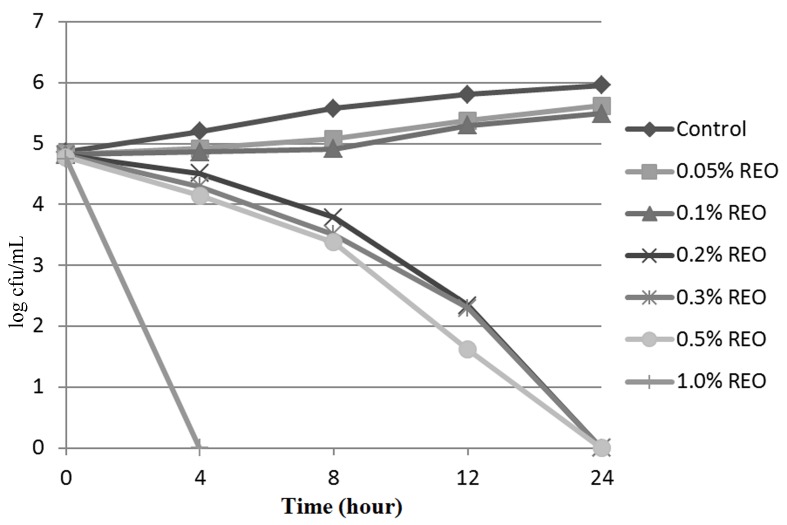
Effect of the REO on the survival of *L. monocytogenes* in TSB.

The antibacterial activities of the REO as determined in TSB units are shown in Figures 1 and 2. In this study, the growth of *S.* Typhimurium and *L. monocytogenes* were inhibited by the REO at 0.2%, 0.3%, 0.5% and 1.0%. Consistent with this result, it has been reported that REO at various concentrations was effective in preventing the growth of food-borne pathogens, such as *S.* Typhimurium (Hammer *et al.*, 1999) and *L. monocytogenes* (Smith-Palmer *et al.*, 1998). In another study, Sagdic and Ozcan (2003) reported that hydrosols of rosemary had no effect on the growth of *S.* Typhimurium. These results showed that the antibacterial activity of REO is affected by the composition of the oil and the strains of bacteria tested. A relationship between the chemical composition of the tested oil and its antimicrobial activity has been reported (Dimitrijevic *et al.*, 2007). Jiang *et al.* (2011) found that the bacteriostatic properties of REO appeared to be associated with its 1.8-cineole and α-pinene contents.

The mean measured headspace compositions of the packages were as follows: 70.4 ± 2.8% N_2_, 27.1 ± 0.9% CO_2_ and 1.3 ± 0.7% O_2_. The gas composition of each type of packaging were nearly constant during the tested storage period. Similar results have been reported by Bingol and Ergun (2011) and Doherty *et al.* (1996). These results may be due to the permeability of the packaging material and the respiration rate of the product. The reduction in the CO_2_ content of the packaged poultry meat was reported to be due to the solubility of CO_2_ in the aqueous phase of the poultry meats (Seydim *et al.*, 2006).

The sensory properties (odor and taste) of the fillets that had been treated using REO at 0.2% were assessed by the panelists, who gave scores above (p < 0.001) the rejection limit (a score of 5), whereas the scores given samples that had been treated using the REO at 0.3%, 0.5% and 1.0% were scored below the rejection limit (p < 0.001). Based on these sensory scores, treatment using 0.2% REO yielded the highest acceptability scores between 5 and 7 days of storage. Due to the very strong odor and taste of the REO when used at a concentration of 0.3%, 0.5% or 1.0% during storage, these concentrations were not utilized in the further experiments. Karabagias *et al.* (2011) evaluated the effect of oregano oil on the odor of lamb meat and found that the sensory data were not generally in agreement with the microbiological data. Ntzimani *et al.* (2010) found that applying REO (0.2%) to cooked chicken produced an acceptable odor and taste. Our findings were similar to these results.

Extracts of volatile compounds obtained from plants are widely used in the food industry due to their ability to inhibit the growth and reduce the number of food-borne pathogens (Kotzekidou *et al.*, 2008). The inhibitory effects of REO on the growth of *S.* Typhimurium and *L. monocytogenes* in poultry fillets are shown in Tables 1 and 2. In this study, treating poultry meat with 0.2% REO did not reduce the populations of *S.* Typhimurium or *L. monocytogenes* (p > 0.05). Our results are agree with those of Fernandez-Lopez *et al.* (2005), who studied cooked meatballs. Farbood *et al.* (1976) showed that 1.0% REO reduced the growth rate of *S.* Typhimurium by 43.2%. Pandit and Shelef (1994) reported that 1.0% REO inhibited the growth of *L. monocytogenes* in a pork liver sausage. Moreover, Shelef *et al.* (1980) demonstrated that Gram-positive bacteria were more sensitive to REO than were Gram-negative bacteria to REO. The differences in susceptibility might be attributed to several factors, including the composition of the REO, the bacterial strain challenged, the pH of the food product and the storage temperature of the product tested. Moreover, the concentration required to achieve a significant antibacterial effect was considerably higher than the MIC that was determined in vitro (Burt 2004). Notably, the growth of the tested pathogens was not affected by air packaging or MAP. No significant differences were found between the groups (p > 0.05). This result is in agreement with that of a previous study published by Vongsawasdi *et al.* (2008).

**Table 1:** Effect of the REO and MAP on the survival of *S.* Typhimurium in the poultry fillets (log cfu/g).

Storage period (day)	Group			
	
	Air	Air + 0.2% REO	MAP	MAP + 0.2% REO
0	4.64 ± 0.07^C^	4.62 ± 0.05^C^	4.66 ± 0.03^C^	4.62 ± 0.04^C^
1	4.78 ± 0.06^C^	4.75 ± 0.05^C^	4.78 ± 0.05^C^	4.75 ± 0.05^C^
3	5.5 ± 0.07^B^	5.53 ± 0.06^B^	5.52 ± 0.06^B^	5.53 ± 0.06^B^
5	5.65 ± 0.07^AB^	5.70 ± 0.08^A^	5.70 ± 0.05^A^	5.66 ± 0.07^AB^
7	5.78 ± 0.06^A^	5.76 ± 0.03^A^	5.76 ± 0.04^A^	5.76 ± 0.06^A^

A–C The mean values within a column indicated using different superscripted letters were significantly different (p < 0.05).

**Table 2:** Effect of the REO and MAP on the survival of *L. monocytogenes* in the poultry fillets (log cfu/g).

Storage period (day)	Group			
	
	Air	Air + 0.2% REO	MAP	MAP + 0.2% REO
0	5.22 ± 0.05^C^	5.20 ± 0.04^D^	5.16 ± 0.04^C^	5.16 ± 0.04^D^
1	5.43 ± 0.04^B^	5.32 ± 0.03^C^	5.33 ± 0.03^B^	5.31 ± 0.03^C^
3	5.47 ± 0.05^B^	5.44 ± 0.04^B^	5.44 ± 0.04^B^	5.44 ± 0.03^B^
5	5.68 ± 0.03^A^	5.64 ± 0.04^A^	5.65 ± 0.06^A^	5.65 ± 0.03^A^
7	5.74 ± 0.04^A^	5.75 ± 0.04^A^	5.71 ± 0.05^A^	5.72 ± 0.05^A^

The mean values within a column indicated using different superscripted letters were significantly different.

The effect of the combining REO treatment with air packaging or MAP on the pH, color parameter and TBA values of the poultry samples are presented in Table 3. In the present study, the pH value was not affected (p > 0.05). During storage, the pH values ranged from 5.92 to 6.07. Similar results for pH values were reported by Cajun *et al.* (2008). Adding REO significantly decreased the L* values (p < 0.001), whereas a significant increase in the a* values (p < 0.001) was observed at 5 and 7 days of storage; the b* values of the samples in the groups remained more similar (p > 0.05) throughout the entire storage period. The results indicated that the MAP conditions had no additional effects on the color-parameter values (p > 0.05). Liu *et al.* (2009) and Sebranek *et al.* (2005) reported similar findings for chicken meat and pork sausages, respectively. Dogan and Dogan (2004) determined that the polyphenol oxidases, which are enzymes found in many plants, are responsible for a browning reaction. However, McCarthy *et al.* (2001) and Estevez *et al.* (2006) found no change in the a* values of frozen pork patties and porcine liver pate, respectively. Balentine *et al.* (2006) stated that adding REO to ground beef caused an increase in the b* (more yellow) value. The differences among the results may be due to differences in the oxidation pattern of oxymyoglobin in the samples, the storage temperature used, the particular muscle type, the light intensity and the meat species (Georgantelis *et al.*, 2007).

**Table 3:** Effect of the REO and MAP on the pH, color-parameter and TBA values of the poultry fillets.

Item	Group	Days of storage				
		
		0	1	3	5	7
pH	Air	5.92 ± 0.04^D^	5.93 ± 0.04^D^	5.97 ± 0.05^C^	6.02 ± 0.09^B^	6.06 ± 0.05^A^
	Air+ 0.2% REO	5.92 ± 0.05^D^	5.93 ± 0.06^D^	5.97 ± 0.04^C^	6.01 ± 0.05^B^	6.06 ± 0.05^A^
	MAP	5.92 ± 0.03^D^	5.93 ± 0.04^D^	5.97 ± 0.06^C^	6.02 ± 0.07^B^	6.07 ± 0.02^A^
	MAP+0.2% REO	5.92 ± 0.04^E^	5.93 ± 0.04 D	5.97 ± 0.06^DC^	6.01 ± 0.02^B^	6.06 ± 0.05^A^
L*	Air	49.46 ± 0.07	49.40 ± 0.10^a^	49.36 ± 0.09^a^	49.33 ± 0.14^a^	49.29 ± 0.04^a^
	Air + 0.2% REO	49.46 ± 0.07^A^	48.54 ± 0.08^b^ ^B^	47.54 ± 0.12^b^ ^C^	47.48 ± 0.12^b^ ^C^	46.52 ± 0.11^b^ ^D^
	MAP	49.46 ± 0.08	49.53 ± 0.09^a^	49.31 ± 0.07^a^	49.45 ± 0.13^a^	49.46 ± 0.12^a^
	MAP+0.2% REO	49.45 ± 0.08^A^	48.51 ± 0.10^b^ ^B^	47.51 ± 0.08^b^ ^C^	47.46 ± 0.12^b^ ^C^	46.56 ± 0.07^b^ ^D^
a*	Air	6.44 ± 0.12	6.46 ± 0.10	6.50 ± 0.11	6.42 ± 0.12^b^	6.48 ± 0.10^b^
	Air+0.2% REO	6.39 ± 0.10^C^	6.40 ± 0.09^C^	7.01 ± 0.22^B^	7.53 ± 0.11^a^ ^A^	7.58 ± 0.12^a^ ^A^
	MAP	6.37 ± 0.08	6.50 ± 0.10	6.44 ± 0.09	6.34 ± 0.07^b^	6.40 ± 0.10^b^
	MAP+0.2% REO	6.40 ± 0.12^C^	6.42 ± 0.10^C^	6.88 ± 0.30^BC^	7.43 ± 0.27^a^ ^AB^	7.60 ± 0.14^a^ ^A^
b*	Air	15.58 ± 0.09	15.40 ± 0.05	15.54 ± 0.04	15.60 ± 0.06	15.57 ± 0.08
	Air+0.2% REO	15.59 ± 0.07	15.42 ± 0.10	15.42 ± 0.11	15.53 ± 0.12	15.67 ± 0.08
	MAP	15.56 ± 0.07^B^	15.43 ± 0.06^AB^	15.39 ± 0.08^AB^	15.51 ± 0.12^AB^	15.66 ± 0.07^A^
	MAP+0.2% REO	15.62 ± 0.11	15.37 ± 0.09	15.41 ± 0.09	15.42 ± 0.13	15.67 ± 0.03
TBA	Air	0.29 ± 0.04^E^	0.51 ± 0.07^a^ ^D^	0.67 ± 0.05^a^ ^C^	0.98 ± 0.05^a^ ^B^	1.26 ± 0.06^a^ ^A^
	Air+ 0.2% REO	0.29 ± 0.04^E^	0.39 ± 0.06^b^ ^D^	0.47 ± 0.07^b^ ^C^	0.62 ± 0.08^c^ ^B^	0.79 ± 0.05^c^ ^A^
	MAP	0.29 ± 0.02^E^	0.41 ± 0.05^a^ ^D^	0.48 ± 0.04^b^ ^C^	0.64 ± 0.04^b^ ^B^	0.81 ± 0.04^b^ ^A^
	MAP+0.2% REO	0.29 ± 0.02^E^	0.33 ± 0.05^c^ ^D^	0.38 ± 0.04^c^ ^C^	0.57 ± 0.07^d^ ^B^	0.75 ± 0.06^d^ ^A^

A, B, C The mean values within a row indicated using different superscripted letters were significantly different p < 0.05).

a, b, c The mean values within a column indicated using different superscripted letters were significantly different (p < 0.05).

The panelists judged the color attributes of the poultry fillets to be similar to those evaluated instrumentally, in which a slight decrease in the brightness of the samples was observed and a slight increase in the redness was noted in REO-treated fillets. The odor and taste of the air-packaged fillet samples became stronger over time due to the increasing level of lipid oxidation; however, the REO- plus MAP-treated samples remained acceptable throughout storage period. Additionally, the MAP-stored samples were more acceptable than were the samples stored with air in terms of odor (data not shown).

The TBA assay is used to measure of the content of MDA, one of the degradation products of lipid hydroperoxides that is formed through the oxidation of unsaturated fatty acids (Patsias *et al.*, 2006). Fillets to which REO was added had significantly lower TBA values (p < 0.001) than did the untreated fillets, and the TBA values increased in all of the groups during the storage period (p < 0.001). The results also showed that adding REO combined with MAP had a strong inhibitory effect on the level of lipid oxidation, whereas the air-packaged fillets had unacceptable TBA values in untreated samples according to the Turkish Standards (TS 2008), with TBA values corresponding to up to 1 mg MDA/kg after the 5^th^ day of storage. It is well known that the development of rancidity as a result of lipid oxidation is one of the major factors limiting the storage life of meat products (Bingol *et al.*, 2012). The beneficial effects of REO that were observed in the present study are in agreement with the results of Yu *et al.* (2002) for turkey and Balentine *et al.* (2006) for ground beef. Additionally, Aruoma *et al.* (1992) reported that carnosic acid and carnosol accounted for more than 90% of the antioxidant properties of a rosemary extract; these compounds are powerful inhibitors of lipid peroxidation. In another study, Chan *et al.* (1997) determined that controlling its autoxidation using rosemary extract reduced the rate of myoglobin color degradation. In contrast, Rojas and Brewer (2007) reported that the addition of REO did not retard the rate of lipid oxidation in cooked pork patties during 8 days of storage. The amount of REO added and the composition of its active compounds may be primarily responsible for the different results (Balentine *et al.*, 2006). According to the Turkish Standards (TS 2008), poultry meat with TBA values of greater than 1 mg MDA/kg is not suitable for human consumption. In the present study, the TBA values were less than 1 mg MDA/kg in the REO plus MAP poultry fillet samples. The O_2_ concentration is the determining factor for lipid oxidation in chicken meat (Seydim *et al.*, 2006) as it is deferred in air packages.

## Conclusion

The results of this study showed that adding 0.2% REO to poultry meat did not reduce the growth rate of *S.* Typhimurium or *L. monocytogenes* at 4 °C during a 7-day storage period. During storage, the presence of REO significantly decreased the L* values and increased the a* values of the fillets. Adding REO in combination with MAP resulted in lower TBA values in the fillets. In conclusion, in a suitable combination, REO can be applied to improve the quality of meat because it retarded lipid oxidation and prevented the development of rancidity; however, REO did not affect the growth of pathogens when used at the lower levels. Further studies should therefore be conducted to determine the commercial level of REO appropriate for different meat products to allow its application by the food industry.
